# P2X7 receptor mediates NLRP3 inflammasome activation in depression and diabetes

**DOI:** 10.1186/s13578-020-00388-1

**Published:** 2020-03-05

**Authors:** Danwen Wang, Hui Wang, Haixia Gao, Heng Zhang, Hua Zhang, Qiuling Wang, Zhiling Sun

**Affiliations:** 1 School of Nursing, Nanjing University of Chinese Medicine, 138 Xianlin Road, Qixia District, Nanjing, 210023 Jiangsu China; 2 Neonatal Intensive Care Unit, Peixian People’s Hospital, Hanyuan Avenue, Xuzhou, 221600 Jiangsu China

**Keywords:** P2X7 receptor, NLRP3 inflammasome, Depression, Diabetes mellitus, Diabetes mellitus with depression, Inflammatory, Baicalin

## Abstract

The increasing prevalence of depression and diabetes mellitus has become a major public health problem worldwide. Studies have shown that people with diabetes are at a high risk of being diagnosed with depression, and diabetes complicates depression treatment by promoting the deterioration of glycemic control, reducing self-care ability and quality of life, and causing severe functional disability and early mortality. Moreover, health deterioration dramatically increases the financial cost of social and health care system. Thus, how to treat depression, diabetes, and diabetes complicated by depression has become one of the world’s urgent concerns. The activation of nod-like receptor family pyrin domain containing 3 (NLRP3) is closely related to mental illness. This finding provides a new perspective for studying depression. NLRP3 plays an important role in the development of diabetes. In this review, we elaborate the definition and epidemiology of depression, diabetes, and diabetic depression and introduce the functional characteristics of an NLRP3 inflammasome and upstream P2X7 receptor. Moreover, related research on NLRP3 inflammasomes and P2X7 receptors is summarized and used as a reference for confirming that the excessive activation of P2X7- NLRP3 leads to the increased release of inflammatory cytokines, such as IL-1β, in depression and diabetes. We provide insights into the P2X7–NLRP3–IL-1β pathway as an important pathological mechanism and novel therapeutic target in diabetes and depression. Given that the P2X7–NLRP3–IL-1β pathway may play an important role in diabetes confounded by comorbid depression, the possibility of intervention with baicalin is proposed.

## Background

Depression is a chronic recurrence and debilitating mental illness and characterized by gloomy mood, anhedonia, low self-esteem, disturbed sleep or appetite, and inattention. Depression, which can last a lifetime, severely impairs an individual’s ability to cope with work or study and even adversely affects daily life activities. Depression is the leading cause of disability in developed countries [1]. According to the World Health Organization, more than 300 Mio. people globally suffered from depression in 2015. Nearly half of them, approximately 4.4% of the world’s population, live in Southeast Asia and the Western Pacific. Nearly 800,000 suicide deaths in 2015 were attributed to depression, and 78% of suicides worldwide occurred in low- and middle-income countries. By 2020, depression is expected to be the second largest social and economic burden affecting more than 10% of the global population. By 2030, depression is expected to be the major cause of the global burden of disease [2].

Along with coronary heart disease, arthritis, cancer, and other diseases related to depression, diabetes mellitus (DM) is a major cause of health deterioration in individuals with depression. DM is a chronic hyperglycemia endocrine disease characterized by dysglycemia. In terms of main pathogenesis, type 1 diabetes mellitus (T1DM) is characterized by insufficient insulin secretion, whereas type 2 diabetes mellitus (T2DM) is characterized by low physiological utilization of insulin. Over the past few decades, the number of diabetics worldwide has steadily increased. By 2014, the number of adult patients was 422 Mio.. More than 300 Mio. adults worldwide have impaired blood glucose regulation, and the risk of diabetes is expected to continuously increase [2]. More than 60% of patients with diabetes come from developing countries. In China, the prevalence of diabetes has risen sharply, from less than 1% in 1980 to 10.9% in 2013. China has the largest number of diabetic patients worldwide, with approximately 109.6 Mio. adults suffering from diabetes, most of whom have T2DM [3]. In 2012, the medical expenditure on diabetes exceeded $322 billion in the United States [4]. Diabetes, especially T2DM, has become a heavy medical care and economic burden for individuals, families, and countries.

Diabetes mellitus with depression (DD), a condition in which depression and diabetes occur simultaneously in the same person, is a serious challenge in clinical diagnosis and treatment. Depression and diabetes may be mutually causal, and depression is an important causative factor for the aggravation of T2DM [5]. When diabetes is associated with depression, the clinical manifestations of depression are often repeated throughout the diabetic’s life [6]. The prevalence of depressive symptoms in diabetic patients is 12%–27%, and a quarter of patients with T2DM are diagnosed with depression [7, 8]. DD impairs the treatment compliance of diabetic patients, significantly increases the risk of complications (diabetic fundus diseases and cerebrovascular diseases), and decreases cognitive function, which may ultimately lead to the deterioration of health outcomes and quality of life and short life span of diabetic patients [9, 10]. As a serious public health problem, DD has increased medical expenses related to diabetes 2.5 times and the total medical expenses four times, seriously aggravating the financial burden of society and national health care system [11].

Despite active treatment by clinicians, the pathogenesis of depression, diabetes, and DD is complicated, making the efficacy unsatisfactory. Therefore, elucidating its pathogenesis is crucial for effective treatment. According to literature, a high comorbidity rate is observed between brain insulin resistance and depression, suggesting a possible common biological substrate [12, 13]. Depression and diabetes are considered low-grade chronic inflammation. It is the biological origin of depression and diabetes that innate immune hyperactivity leads to cytokine-mediated inflammatory response. Inflammasomes, especially NLRP3, play an important role in innate immunity and are activated in chronic inflammatory states. Herein, we summarize the effect of the NLRP3 inflammasome and P2X7 receptor in DM and depression, respectively, and deduce their pathological roles in DD. Moreover, the possibility of treating DD with baicalin as a complementary and alternative approach is proposed.

## NLRP3 inflammasome and upstream P2X7 receptor

Inflammasomes are polyprotein complexes composed of an apoptosis-associated speck-like protein containing CARD (ASC), precursor cysteinyl aspartate specific proteinase-1 (caspase-1), and nod-like receptor (NLR) family proteins, which are induced by pathogen-associated molecular patterns (PAMP) or danger-associated molecular patterns (DAMP) [14]. Inflammasomes play important roles in innate immunity by activating caspase-1, promoting the maturation and secretion of interleukin-1 beta (IL-1β), and producing corresponding mature cytokines. Moreover, inflammasomes regulate the inflammatory necrosis (pyroptosis) of cells, which induces cell death under the pathological conditions of inflammation and stress. The NLRP3 inflammasome, which is composed of NLRP3 as the recognition receptor, ASC as the adapter, and caspase-1 as the effector protein, is currently one of the most widely studied members of the NLR family. Relevant studies on its mechanism of action and the role of the upstream protein P2X7 receptor in depression and diabetes have been conducted.

Purine signaling systems, such as P2 receptors, play an important role in regulating neurotransmission, neurotransmitter activity, and some diseases [1, 15]. P2X7 receptors, which belong to the ligand-gated ion channel P2X subfamily of purinergic P2 receptors, can be activated by high concentrations of extracellular adenosine 5′-triphosphate (ATP). P2X7 receptor plays an important role in mediating innate immune response by regulating the expression of the proinflammatory cytokines of the IL-1 family [16–18].

P2X7 receptors are widely present in tissues and cells, such as neutrophils, which are activated by ATP to induce inflammation [19]. In the central nervous system (CNS), P2X7 receptor is mainly expressed in microglia cells but its expression level is low in astrocytes and some neurons [20]. β cells are the only cells that express vesicular nucleotide transporters (VNUTs), which can accumulate ATP in the pancreas and store and release ATP from insulin granules [21]. The pancreatic ducts release ATP through multiple systems to increase extracellular ATP level in the exocrine pancreas [22]. P2X7 receptors are activated by ATP to induce overactivation of NLRP3 inflammasome.

## NLRP3 inflammasome and upstream P2X7 receptor as biological substrates for depression and diabetes

### Role of the NLRP3 inflammasome in depression

Hypotheses about the pathogenesis of depression are numerous. One of these hypotheses is that inflammation and immunity are important factors affecting the occurrence and development of mood disorders. Neuroinflammation is caused by the excessive secretion of inflammatory cytokines in the brain and is considered one of the important mechanisms of depression [23]. Chronic uncontrolled stress is a major cause of depression, which activates the innate immune system in the peripheral nervous system and CNS, and leads to the activation of caspase-1 by the NLRP3 inflammasome interacting with ASC and participates in the production of inflammatory cytokine processes, such as IL-18 and IL-1β [24, 25]. Acute and chronic stress leads to increased levels of proinflammatory cytokines, including IL-1β, tumor necrosis factor (TNF), and IL-6 [26]. Inflammatory responses in the hippocampus, especially the enhancement of IL-1β signaling, may lead to the development of depressive symptoms [27]. The expression levels of NLRP3 inflammasome mRNA and IL-1β are significantly increased in the brains of depressed mice induced by lipopolysaccharide (LPS), suggesting that IL-1β and NLRP3 inflammasome are the mediators of inflammation between stress and depression [28]. NLRP3 inflammasome, ASC, and caspase-1 expression levels were enhanced in mice with chronic unpredictable stimulation and blocked in NLRP3 knockout [29].

Recently, an interesting study has shown that predictable chronic mild stress, the routine stress experienced in daily life, facilitates recovery from LPS-induced depressive behavior by inhibiting the overactivation of NLRP3 inflammation and IL-1β maturation [30]. A study on the relationship between gut microbiota and depression showed that the depressive-like behavior of recipient mice induced by chronic unpredictable stress can be significantly alleviated by transplanting an NLRP3 knockout microbiota [31]. The key mechanism is the inflammatory response triggered by the Nrf2/NLRP3 signaling pathway, which has been demonstrated in the study of PM2.5 pollution-induced depression [32]. The activation of NLRP3 inflammasomes plays a key role in the influence of early adverse experience on adult depression. Therefore, chronic antidepressant treatment can attenuate depression-like disturbances in the hippocampus by inhibiting the activation of NLRP3 inflammasomes [33]. NLRP3 inflammasomes are considered important mediators of depression induced by immune activation during stress exposure. Psychological stress caused by adverse social interactions and nonpathogenic risk signals may activate the inflammasome pathway [34]. The mechanism of depression caused by psychological stress may be to increase the sensitivity of innate immune cells, such as microglia and monocytes, to activate NLRP3 inflammasomes, which further promote the release of proinflammatory cytokines [13, 35]. The suicide ideation of depressive patients is positively correlated with the level of IL-1, suggesting that IL-1 and NLRP3 inflammasomes are mediators of depression induced by psychological stress [36]. However, how psychological stress causes NLRP3 inflammasome formation is still unclear.

Pharmacotherapy studies have shown that some antidepressants, such as amitriptyline, a common tricyclic antidepressant, inhibit the activation of inflammasomes and reduce IL-1β production and the expression of NLRP3 and caspase-1 genes in depressed patients [37]. In inflammatory cytokines, serum IL-1β level is reduced after antidepressant treatment, whereas serum TNF-α and IL-6 levels are unchanged, suggesting that IL-1β level is correlated with the effectiveness of antidepressant treatment. However, IL-1β is an inflammatory cytokine produced by activated NLRP3 inflammasome; thus, the levels of IL-1β and NLRP3 are negatively correlated with the antidepressant effect, that is, high levels of NLRP3 and proinflammatory cytokines show unsatisfactory antitherapeutic effect [38].

Normal levels of glucocorticoids (cortisol or corticosterone) exhibit anti-inflammatory and immunosuppressive effects. Excessive glucocorticoid production and glucocorticoid resistance are associated with depression and possibly leads to an inflammatory state by losing the inhibition of immune cells. In mice treated with chronic unpredictable mild stress (CUMS), cortisol in the serum of an NLRP3 gene knockout mice decreased sharply, 5-hydroxytryptamine (5-HT) and norepinephrine (NE) are significantly increased, and brain-derived neurotrophic factor level in the hippocampus is not decreased [39, 40]. Therefore, NLRP3 plays an important role in regulating the onset and development of depression.

### Role of P2X7 receptor in depression

In preclinical studies, P2X7 receptor signal changes mediate depression-like behaviors. For example, hippocampal P2X7 receptors are activated by a large amount of extracellular ATP during acute immobilization stress, which induces the activation of NLRP3 and leads to the release of inflammatory cytokines [25]. Some P2X7 selective antagonists that can penetrate the blood–brain barrier (BBB) exhibit therapeutic effects on depression in a chronic stress model by inhibiting the activation of P2X7–NLRP3–IL-1β pathway, reducing the immune inflammatory effect of microglia cells and the neuroinflammation of CNS [41]. In the CUMS paradigm, the activation of P2X7–NLRP3–IL-1β pathway is induced by increased extracellular ATP levels in the hippocampus and prolonged adverse stress, resulting in the persistence of depressor-like behaviors [18]. CUMS-induced depression-like behavior is eliminated in P2X7-gene knockout and chronic administration of P2X7 antagonists, such as bright blue G (BBG) and A438079 [18].

**Fig. 1:**
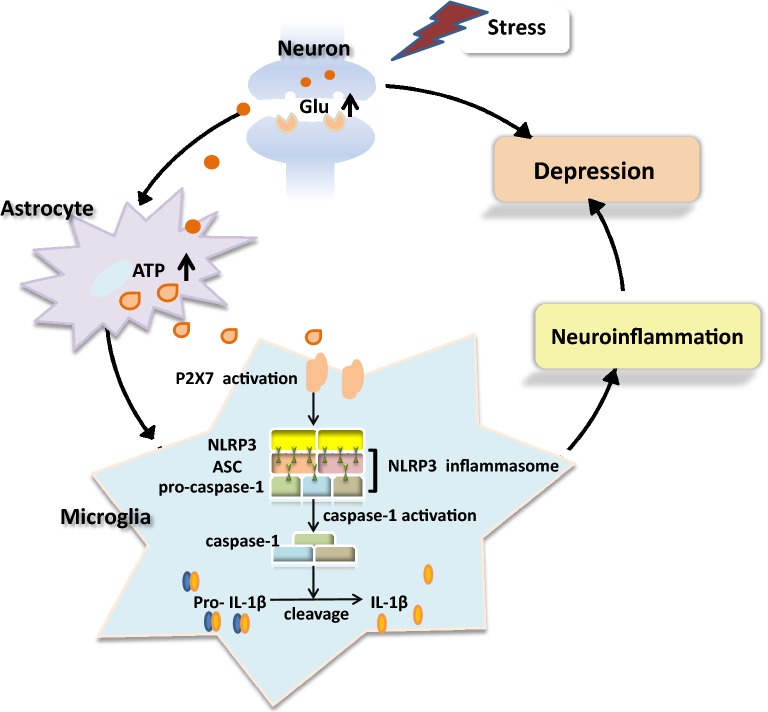
Pathological mechanism of depression induced by the P2X7–NLRP3–IL-1β pathway. Stress stimulates the production and release of excessive Glu at the synaptic terminals of neurons, promoting astrocytes to release a large amount of ATP. In the microglia, P2X7 receptors are activated by excessive extracellular ATP levels, which lead to cascade reactions and induce the overactivation of NLRP3 inflammasomes. Activated caspase-1 induces the maturation and release of IL-1β, which triggers neuroinflammation that can lead to depression

Adverse psychological stress increases extracellular ATP level in the brain and promotes the activation of P2X7 receptors by microglia, thus inducing CNS neuroinflammation and finally leading to depression [42, 43]. Stress induces excessive glutamate (Glu) production by nerve terminals, which not only produce excitotoxicity but also stimulate astrocytes to secrete a large amount of ATP; thus, stress leads to a cascade of reactions involving P2X7 receptors and increases IL-1β level in the brain [44]. In addition, excitotoxicity induced by the abnormal increase in Glu level may directly or indirectly downregulate the production of BDNF and its neuroplasticity, eventually leading to the occurrence of depression [45]. When subjected to adverse stimuli, P2X7 ion channels are activated to induce the release of NLRP3-induced proinflammatory factors, such as IL-1β, leading to neuroinflammation of the CNS and increasing incidence of depression [46, 47]. Thus, the activation of the P2X7–NLRP3–IL-1β pathway can induce the onset of depression (Fig. 1).

However, P2X7 receptor expression is reduced in several subregions of the hippocampus after acute and chronic constraint stress, suggesting that reduced ATP–P2X7 receptor signal intensity increases the incidence of depression [48]. Therefore, the role of P2X7 receptor-mediated NLRP3–IL-1β pathway in depression must be clarified.

### Role of NLRP3 inflammasome in DM

T2DM is not only a chronic metabolic disease characterized by metabolic disorders and hyperglycemia but also an autoimmune inflammatory disease caused by metabolic stress. DM is considered a parainflammation, and inflammatory response plays an important role in the insulin resistance of T2DM [14, 49, 50]. Notably, the activation of NLRP3 inflammasomes not only induces depressive symptoms but also impairs glucose tolerance in the liver and brain of CUMS mice. This condition can restore glucose tolerance by improving insulin signaling and inhibiting the activation of NLRP3 inflammasomes [51]. Preclinical studies have found that chronic hyperglycemic metabolic stress may induce the increased secretion of NLRP3-dependent IL-1β, possibly leading to the development of T2DM and that reducing the production of IL-1β may lessen the incidence of T2DM in animal models [44, 52]. Therefore, the inflammatory mechanism in the pathogenesis of T2DM may be due to the activation of NLRP3 inflammasomes, which in turn produce IL-1β. A known proinflammatory cytokine, IL-1β accelerates the development of insulin resistance, stress, depression, and CNS dysfunction [53]. IL-1β may cause pancreatic β cell death and dysfunction, thus accelerating the development of insulin resistance. Increased levels of IL-1β and impaired β cell function lead to the onset of T2DM by inhibiting the insulin signaling pathway. Therefore, NLRP3 and IL-1β levels may be used as the major predictors of T2DM [54, 55].

Studies have shown that by inhibiting the activation of NLRP3 inflammasome, some hypoglycemic drugs exhibit antidepressant effects, whereas antidepressants exhibit the effect of regulating glucose metabolism. Glibenclamide is not only a sulfonylureas antidiabetic drug but also an effective inhibitor of NLRP3 inflammasome, which can regulate metabolic disorders caused by chronic stress and alleviate depressor-like behaviors by inhibiting the production of inflammasomes in the circulatory system [56, 57]. Fluoxetine, a selective serotonin reuptake inhibitor, acts as an antidepressant and blood glucose regulator by inhibiting NLRP3 inflammasome to reduce inflammatory cytokines and regulate insulin signaling [58, 59]. We concluded that NLRP3 inflammasome is related to the occurrence and progression of T2DM.

The neuroendocrine immune network, especially in the hypothalamic–pituitary–adrenal axis (HPA), is closely associated with T2DM. Glucocorticoid secreted by the HPA axis adversely regulates blood glucose homeostasis by disrupting the insulin signaling pathway and glucose metabolism [60]. Glucocorticoids increase blood glucose level by stimulating the gluconeogenesis of the liver and inhibiting glucose uptake by tissues. Increase in glucocorticoid secretion because of HPA axis dysfunction can directly antagonize insulin effect, inhibit insulin release, destroy glucose metabolism, and reduce insulin sensitivity. NLRP3 is considered a potential target gene for glucocorticoid receptor, and the level of NLRP3 mRNA in the hippocampus is increased by exposure to exogenous glucocorticoids [61–63]. The hyperactivity of the HPA axis in CUMS rats induces the activation of NLRP3 inflammasomes and the maturation of IL-1 by promoting corticosterone secretion in the peripheral nervous system and CNS, leading to an impaired insulin signaling pathway. Therefore, HPA dysfunction may exist in depression and T2DM, but its mechanism still needs to be further studied.

### Role of P2X7 receptor in DM

**Fig. 2:**
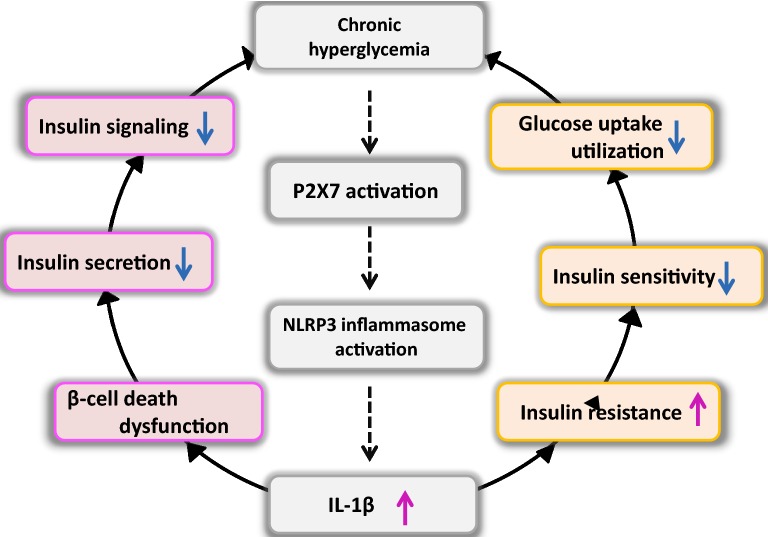
Pathological mechanism of DM induced by the P2X7–NLRP3–IL-1β pathway. Chronic hyperglycemia stimulates the activation of the P2X7–NLRP3 inflammasome, resulting in the excessive release of proinflammatory factor IL-1β. By inducing pancreatic β cell dysfunction and death, IL-1β can reduce insulin secretion, impair the insulin signal pathway, and aggravate hyperglycemia. In addition, IL-1β accelerates the development of insulin resistance, and with the decrease of insulin sensitivity, the cellular uptake and utilization of glucose are reduced, hyperglycemia is further aggravated, and diabetes is finally induced

In addition to depression, P2X7 receptor is closely related to the occurrence of diabetes and some diabetes comorbidities [64]. P2X7 receptors regulate insulin secretion by participating in pancreatic β cell function, and the abnormality of P2X7 function causes the imbalance in energy homeostasis and increases the accumulation of adipose tissues, which have potential effects on metabolic diseases [65]. P2X7 receptor knockout and P2X7 receptor inhibitor BBG can prevent diabetes induced by streptozocin [66]. The overexpression of P2X7 receptor exhibits adverse effects on diabetic retinopathy, diabetic neuropathy, and diabetic nephropathy [67–69], and the impaired activation of the P2X7 receptor signaling pathway in T1DM may affect the function of osteoblasts and bone health [70, 71]. When glucose metabolism is disordered, excessive extracellular ATP level stimulates macrophages in the pancreas and adipose tissues, thus prompting P2X7 receptors to activate NLRP3 inflammasomes [72]. The increased level of NLRP3-dependent IL-1β induces and aggravates T2DM through β cell death and insulin resistance. Therefore, the activation of NLRP3 inflammasomes by P2X7 receptors may be an important pathogenesis of diabetes (Fig. 2).

Although P2X7 receptors regulate the function and survival of β cells and promote insulin release, whether P2X7R signatures are the same in human and rodent β cells remains unknown [73].

## Role of P2X7 receptor and NLRP3 inflammasome in DD

The obvious characteristics of T2DM are insulin resistance and recurrent hyperglycemia, which can lead to systemic inflammatory response. Increased release of inflammatory mediators in depression may affect insulin sensitivity and pancreatic cell function, thus accelerating the development of T2DM. Increasing evidence suggests that inflammatory response mediated by the overactivated cytokines of innate immunity is an important component of the biological mechanism shared by depression and T2DM [74]. A In rats, chronic high-fat diet (HFD) activates the innate immune system and increases the release of inflammatory cytokines, leading to T2DM and depression. Blocking P2X7 receptor and NLRP3 inflammasome activation can restore brain homeostasis and significantly alleviate depression in HFD rats [75]. NLRP3 inflammasome activation and IL-1β production induced by adverse stress not only induce depressive-like behavior but also impair the insulin signaling pathway [76]. Insulin resistance is associated with depression and T2DM, possibly leading to DD [6, 77]. The serum levels of IL-1β and homoeostatic model assessment-insulin resistance increase in patients with depression, and neuroinflammation and insulin resistance induced by metabolic imbalance can induce depressive disorder [78, 79]. The activation of NLRP3 inflammasomes to produce IL-1β leads to the coexistence of depressive-like behavior and insulin resistance in CUMS mice [57].

**Fig. 3:**
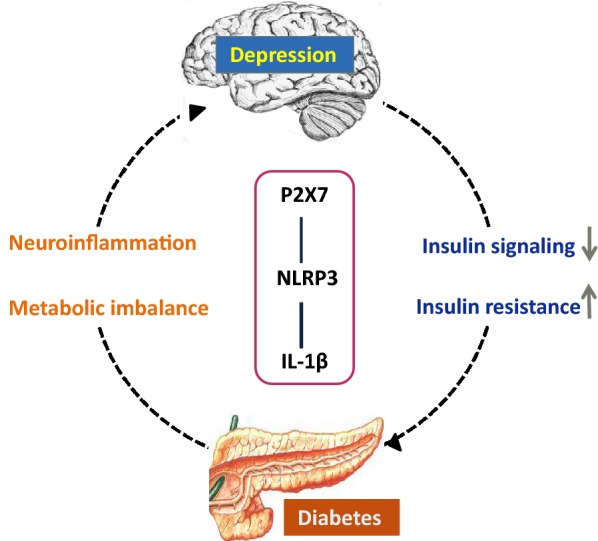
Pathological mechanism of DD induced by the P2X7–NLRP3–IL-1β pathway. The activation of P2X7-NLRP3 inflammasome leads to systemic immune responses, inducing depression and diabetes. NLRP3-dependent IL-1β may lead to insulin resistance and impaired insulin signal in depressed patients, thus promoting the occurrence of diabetes. Moreover, the P2X7–NLRP3–IL-1β pathway aggravates metabolic imbalance and triggers the neuroinflammation of the central nervous system, rendering diabetic patients prone to comorbid depression

Patients with diabetes suffer from huge psychosocial pressure, which may be related to strict diabetes diet and exercise self-management, excessive medical expenses, dysregulation of blood glucose, and impaired quality of life [9]. The psychological stress of diabetic patients can induce P2X7–NLRP3 inflammasome activation, thus triggering depression. The psychological and behavioral effects of depression aggravate the dysglycemia of T2DM, resulting in serious adverse effects on the physical and mental health of patients. Thus, the activation of P2X7–NLRP3–IL-1β pathway may be the pathogenesis of DD (Fig. 3).

## Thoughts on baicalin in the treatment of DD

The adverse reactions of drug therapy further promote and aggravate the development of DD. With the increase in the number of patients with DD, clinical investigation and therapeutic intervention studies are increasing, but reports on preclinical studies on biological origin, especially on the P2X7–NLRP3–IL-1β pathway, are few. In addition, the administration of antidepressant pharmacotherapy is considered an intermediary between depression and T2DM [11]. Some antidepressants may cause glucose and lipid metabolism disorders, such as weight gain and dyslipidemia, leading to the severe deterioration of patients with T2DM [80, 81]. Depression and T2DM drug treatments have unsatisfactory outcomes in clinical practice mainly because the adverse side effects of drugs and the high rate of secondary failure, resulting in a significant decline in treatment compliance. Thus, finding effective drugs to treat DD is urgent.

As a complementary and alternative method, traditional Chinese medicines with anti-inflammatory and anti-hyperglycemia effects are increasingly attracting the interest of patients with diabetes and medical staff. The Radix Scutellariae plant is a common clinical Chinese medicine and is first recorded in Shennong herbal classic, a great work on traditional Chinese medicine. Its roots exert several effects, such as clearing heat, drying dampness, reducing fire and detoxifying, hemostasis, and tocolysis. Baicalin, the main component of the Radix Scutellariae plant, is a kind of polyphenol isolated from *Scutellaria baicalensis* root. Baicalin can penetrate BBB, has strong biological activity, and has been widely used in the clinical treatment of infectious and inflammatory disorders [82]. Preclinical studies have shown that baicalin exhibits antidepressant effects by inhibiting the GSK3β/NF-κB/NLRP3 signaling pathway and downregulating overactivated HPA axis [83–87]. Moreover, baicalin can mitigate obesity and insulin resistance during diabetes treatment by activating the AKT/AS160/GLUT4 pathway and increasing the insulin sensitivity of lipocytes [88, 89]. However, its regulatory effect on the P2X7–NLRP3–IL-1β pathway in DD has not been reported. Therefore, its pathogenesis and the preventive and therapeutic effects of traditional Chinese medicines, such as baicalin, must be further explored.

## Conclusion

Depression and DM are clinically predisposing diseases with a large number of patients, and the incidence of depression in diabetic patients is increasing. Comorbidity is one of the main challenges faced by medical and scientific communities. It is a clinical situation in which two or more diseases occur simultaneously in the same patient. Diabetic patients are the high-incidence population of depression. These diseases interact and aggravate each other. DD impairs patients’ adherence to therapy and increases the risk of serious short- and long-term complications, which may eventually lead to amputation, cognitive impairment, decreased quality of life, and premature death. Therefore, diabetes confounded by depression is harmful to patients. As mentioned above, the P2X7-mediated activation of NLRP3 inflammasome plays an important role in the onset and progression of diabetes and depression. Therefore, we believe that cytokine-mediated inflammatory response induced by innate immune hyperactivity may be the biological source of DD. The role of the P2X7–NLRP3–IL- 1β pathway in DD needs to be studied clearly. We expect that baicalin can effectively treat DD by regulating the P2X7–NLRP3–IL-1β pathway.

## Data Availability

Not applicable.

## Abbreviations

DM: Diabetes mellitus
T1DM: Type 1 diabetes mellitus
T2DM: Type 2 diabetes mellitus
IL-1β: Interleukin-1 beta
ASC: Apoptosis-associated speck-like protein containing CARD
Caspase-1: Cysteinyl aspartate specific proteinase-1
NLR: Nod-like receptor
NLRP3: Nod-like receptor family pyrin domain containing 3
PAMP: pathogen-associated molecular patterns
DAMP: Danger-associated molecular patterns
ATP: Adenosine 5′-triphosphate
VNUT: Vesicular nucleotide transporter
TNF: Tumor necrosis factor
LPS: Lipopolysaccharide
CUMS: Chronic unpredictable mild stress
5-HT: 5-Hydroxytryptamine
NE: Norepinephrine
Glu: Glutamate
BBB: Blood–brain barrier
CNS: Central nervous system
BBG: Bright blue G
HPA axis: Hypothalamic–pituitary–adrenal axis
HFD: High-fat diet
STZ: Streptozocin
DD: Diabetes mellitus with depression
